# Acute Kidney Injury During Sepsis and Prognostic Role of Coexistent Chronic Heart Failure

**DOI:** 10.3390/jcm14030964

**Published:** 2025-02-03

**Authors:** Jens Soukup, Rainer U. Pliquett

**Affiliations:** 1 Department of Anesthesiology, Intensive Care Medicine and Palliative Care, Medical University Lausitz—Carl Thiem, Thiemstr. 111, 03048 Cottbus, Germany; 2 Department of Nephrology, Hypertensiology and Geriatry, Elblandklinikum Riesa, 01589 Riesa, Germany; rpliquett@endothel.de; 3 Department of Internal Medicine II, Medical Faculty, Martin-Luther University Halle-Wittenberg, 06120 Halle, Germany

**Keywords:** sepsis, kidney injury, heart failure

## Abstract

**Background:** The recently updated definition of sepsis considers pathophysiologic mechanisms to guide initial therapy. Clearly, generalized recommendations for sepsis therapy may be limited by pre-existing multimorbidity in addition to sepsis-related multi-organ failure. In particular, a recommendation regarding fluid rescue therapy may require adequate cardiac function and/or the absence of sepsis-induced cardiomyopathy. In all sepsis patients with compromised cardiac function or sepsis-induced cardiomyopathy, a patient-specific therapy regimen is required to prevent pulmonary edema and early death. Similarly, in sepsis, acute kidney injury with or without pre-existing chronic kidney disease requires attention to be paid to excretory renal function to avoid hypervolemia-mediated acute heart failure. In addition, hyponatremia related to intravascular hypovolemia may be explained by vasopressin stimulation. However, hypothetically, vasopressin hyporesponsiveness may contribute to sepsis-related acute kidney injury. In this review, relevant cardiorenal pathomechanisms will be assessed in the context of sepsis therapy. **Conclusions:** In conclusion, therapy for sepsis with acute kidney injury has to take cardiac comorbidity, if present, into account. The extent to which vasopressin hyporesponsiveness aggravates sepsis-mediated hypovolemia and renal insufficiency should remain a subject of further study.

## 1. Introduction

The sepsis-3 definition reflects a better understanding of its newly discovered pathophysiological mechanisms [1,2]. While advances in the diagnosis and treatment of this disease have highlighted the importance of optimized initial therapy, breakthrough innovations in this area remain elusive. Sepsis remains a leading cause of death worldwide [3]. It also represents an increasing socioeconomic burden due to the large number of patients, the high cost of hospitalization and the long follow-up care that is sometimes required to compensate for the consequences of sepsis. In the United States, sepsis treatment costs between USD 20,000 and USD 50,000 per patient, making it one of the most expensive hospital treatments, costing more than $20 billion annually. Similar costs are reported from Europe, where treatment costs per patient range from EUR 7500 to EUR 27,000 [4].

Kidney failure, in the form of acute kidney injury (AKI) and pre-existing chronic kidney disease (CKD), and heart failure, which can be acute (acute heart failure, AHF) or chronic (chronic heart failure, CHF), are individual risk factors for poorer outcomes [5,6]. Sepsis can cause tubular and glomerular damage, worsening pre-existing CKD through successive episodes of sepsis-related AKI. At the same time, renal insufficiency worsens the prognosis of sepsis patients by limiting the excretion of inflammatory mediators and drugs, which can increase drug toxicity and complicate the management of fluid and electrolyte balance. As a consequence of renal failure, fluid overload can lead to the deterioration of other organ systems, particularly the cardiovascular system.

Heart failure can be pre-existing or sepsis-related, including reduced systemic perfusion and inadequate oxygen delivery to vital organs. This increases susceptibility to further septic complications such as shock and multiple-organ failure [7]. The simultaneous presence of renal insufficiency and heart failure in sepsis forms so-called cardiorenal syndrome type 5, which, according to the Ronco classification, represents a complex pathophysiological interface [8]. The cardiorenal syndrome complicates the treatment of sepsis, as, for example, sepsis-induced hypotension is exacerbated by severe heart failure and edema due to low albumin levels and is further exacerbated by acute kidney injury (AKI) or chronic kidney disease (CKD). This combination of cardiac and renal insufficiency increases the risk of treatment failure in sepsis. In the early stages of sepsis, individualized therapy is critical to balance aggressive hydration with the avoidance of volume overload, especially in patients with impaired cardiac and renal function.

The aim of this work is to understand the individuality of cardiac and renal failure and their combination, in order to elucidate the principles of personalized sepsis therapy based on the relevant pathophysiological mechanisms.

## 2. Sepsis and the Development of Organ Insufficiencies

Sepsis is caused by an infection, usually in the respiratory tract, abdominal cavity and urinary tract, that results in an inappropriate, dysregulated immune response to pathogenic microorganisms. The sepsis-3 definition addresses this dysregulated immunologic response as a main cause for the development of sepsis [2,9].

Infections with pathogenic microorganisms trigger an inflammatory response with pro- and anti-inflammatory processes, which are usually acting in a localized and regulated fashion. Balanced pro- and anti-inflammatory processes are intended to protect the human organism from a systemic spread of infection. Depending on the pathogenicity of the microorganisms, the immune response can disrupt the immunological balance and may lead to an uncontrolled, excessive response. Microorganisms or their molecular components, such as Gram-negative bacterial lipopolysaccharides (LPS) or beta-D-glucan of fungal organisms, are recognized as pathogen-associated molecular patterns (PAMPs). These PAMPs are identified by extracellular and intracellular pattern recognition receptors (PRRs), including Toll-like receptors (TLRs) and NOD (nucleotide-binding oligomerization domain)-like receptors, which trigger the primary activation of the innate immune system. The binding of PAMPs and damage-associated molecular patterns (DAMPs), including heat shock proteins, adenosine triphosphate (ATP) and high-mobility group protein B1, to extracellular and intracellular pattern recognition receptors triggers a cascade of signal transduction pathways that promote the production of cytokines. In particular, the activation of the transcription factor nuclear factor kappa B (NF-κB) and mitogen-activated protein kinase (MAPK) signaling pathways is enhanced by PRRs, leading to the excessive production of proinflammatory cytokines, including tumor necrosis factor (TNF) alpha, interleukin (IL)-1β and IL-6. These cytokines induce n increased expression of adhesion molecules (e.g., ICAM-1, VCAM-1) on endothelial cells, which promote the adhesion of leukocytes to the endothelium, thus enabling the migration of immune cells into the tissue. At the same time, these mediators may increase vascular permeability, leading to leakage of fluid and proteins into the extravascular space, resulting in edema and reduced connective-tissue perfusion [10,11,12]. The impaired microcirculation is further exacerbated by the activation of the coagulation cascade, leading to the formation of microthrombi. In conjunction with endothelial damage and systemic inflammation, microthrombi may promote disseminated intravascular coagulation (DIC), thus impairing the microcirculation of organs, triggering organ hypoperfusion. In critical cases, this microcirculatory disturbance leads to a complete loss of organ function [12,13]. Mitochondrial dysfunction represents another central pathomechanism contributing to organ dysfunction caused by sepsis. Cytokines such as TNFα and IL-1β can directly affect mitochondrial function by destabilizing mitochondrial membranes and inhibiting oxidative phosphorylation. This mitochondrial stress reduces ATP production in the affected cells, thereby worsening cellular energy deficiency. Specifically, the heart, the liver, the kidneys and the brain are vulnerable to this mitochondrial dysfunction, as energy deficiency impairs the maintenance of basic cellular functions and repair mechanisms.

There are also relevant pathological changes on the level of arterioles, which, ultimately, may lead to organ dysfunction or organ failure [14,15]. Two phases can be identified: the hyperdynamic phase, characterized by increased cardiac output and reduced systemic vascular resistance (SVR), and the hypodynamic phase, with reduced cardiac output and normal or increased SVR. Pronounced vasodilatation, which leads to a significant decrease in blood pressure, is primarily triggered by the LPS- and TNFα-induced activation of inducible nitric oxide synthase (iNOS) in endothelial cells, which catalyzes the conversion of L-arginine to nitric oxide (NO). In sepsis conditions, the activity of iNOS outpaces that of the constitutive, endothelial NO synthase (eNOS), resulting in increased NO production and, consequently, in pronounced vasodilation. This vasodilation contributes to lowering blood pressure by reducing SVR in the hyperdynamic phase of sepsis [16].

In addition, prostaglandins, which play an important role in the inflammatory response, are synthesized and released by endothelial cells during sepsis. In the hyperdynamic phase of septic shock, the prostaglandins PGE and PGI cause cAMP-mediated vasodilation through the activation of prostaglandins EP_2_- and EP_4_- and IP-receptors, which lowers vascular resistance and leads to hypotension. In addition, impaired microcirculation and increased vascular permeability worsen organ function. In the hypodynamic phase, vasoconstrictive prostaglandins such as thromboxane A_2_ (TXA_2_) promote calcium-mediated vasoconstriction via TP receptors. This phase is characterized by reduced cardiac output, tissue hypoperfusion and impaired microcirculation. In addition, prostaglandins exacerbate vascular dysfunction in both phases through their proinflammatory effects. Balance between vasodilatory (e.g. PGE_2_, PGI_2_) and vasoconstrictive (e.g. TXA_2_) prostaglandins is crucial to enabling hemodynamic changes in septic shock and offers the possibility of targeted pharmacological modulation of these signaling pathways [1].

Finally, regional hypoxia may occur, resulting in a shift in acid–base balance and an increase in lactate tissue levels. There is a high level of scientific evidence that lactate with a cut-off of ≥2 mmol/L in combination with hypotension (MAP ≤ 65 mmHg) is associated with an individual’s risk of death. In fact, as lactate rises, the risk of death increases linearly. For example, the odds ratio for hospital mortality increases from 1.4 (95% CI, 1.35–1.45) to 3.03 (95% CI, 2.68–3.45) when serum lactate increases from 2 to 10 mmol/L [15].

In addition, despite adequate oxygen supply at the whole-body level, inadequate oxygen utilization at the cellular level may occur, which is called “cytopathic hypoxia” [17]. This form of hypoxia may lead to an impaired cellular mitochondrial respiration chain and impaired cell function. Experimental studies have shown that endotoxins may uncouple mitochondrial ATP synthesis by uncoupling cytochrome c oxidase in the respiratory chain. This process reduces ATP production, regardless of the availability of oxygen, leading to further deterioration of cellular energy supply and, thus, organ dysfunction. In the later stages of sepsis, immunological dysregulation, clinically defined as immune paralysis, may occur. This phase is characterized by a reduced ability of the body to mount an effective response to new infections. This is caused by the overactivation of regulatory T cells and suppression of other subsets of T lymphocytes and macrophages. This immunosuppression leads to increased susceptibility to secondary infections, which, in turn, may increase the risk for further progression of sepsis.

## 3. Renal Failure and Sepsis

As for the prevalence of renal failure in sepsis, up to 60% of patients with sepsis experience an AKI, and sepsis patients account for approximately 50% of all AKI cases in the critical care setting [18,19]. Furthermore, sepsis-associated AKI (SA-AKI) has a worse prognosis than AKI due to other causes [20,21]. Approximately 40% of patients with moderate to severe SA-AKI who recovered before hospital discharge and had a slightly poorer 3-year survival rate (28% mortality) in comparison to patients without SA-AKI (23% mortality). Those patients who did not recover had the poorest prognosis (44% mortality) [22]. However, even in patients whose renal function recovered, AKI frequently recurred during the initial recovery period [23]. Finally, the time to recovery from SA-AKI may also be a prognostic parameter [24].

Thus, renal failure in sepsis is not merely a consequence of systemic hypoperfusion, but it results from a complex interplay of different pathophysiological mechanisms triggered by a dysregulated inflammatory response. In the early stages of sepsis, the central mechanisms include local inflammation of the nephrons, glomerular damage due to hypoxia–reperfusion injury, oxidative stress, the cytotoxic effects of cytokines and chemokines, and the apoptosis of tubular and mesangial cells [25], leading to impaired renal function [26,27].

In addition, sepsis may lead to the activation of endothelial cells, resulting in the increased release of proinflammatory cytokines and in the formation of microthrombi. As a result, the microvascular oxygen supply to the kidneys is impaired, which is further exacerbated by the formation of endothelial leaks. In various clinical sepsis situations, the interaction of DAMPs and PAMPs with pattern recognition receptors on immune cells may further initiate a systemic inflammatory response, leading to a complex cascade of dysregulated immune responses. Likewise, bacterial components such as LPS reduce the renotubular expression of megalin and cubilin, which inhibit tubular protein reabsorption [26].

In addition to these cellular and molecular mechanisms, hemodynamic changes during sepsis may lead to cytotoxic damage to renal cells, thus further exacerbating renal function. The specific pathomechanisms of cytotoxic damage include apoptosis, necrosis, necroptosis and pyroptosis.

As arterial perfusion of the kidneys is central to renal function, microcirculatory disorders from the activation of endothelial cells, promoting the expression of adhesion molecules such as P-selectin and ICAM-1, represent an upcoming, potentially treatable focus in sepsis. Adhesion molecules promote the adherence of leukocytes and platelets to the endothelium, possibly leading to further disturbances in the microcirculation as well as to the intensification of inflammatory reactions due to other causes.

The sepsis response also leads to heterogeneous microvascular blood flow in the kidneys due to reduced capillary density and uneven distribution of blood flow [28]. This microcirculatory disturbance induces regional hypoperfusion, resulting in local hypoxia and an increased inflammatory response [29]. In these areas, there is intense leukocyte infiltration, increased local activation of coagulation and microthrombus formation, thus further exacerbating hypoperfusion and increasing renal cell injury and cell death [26,29,30].

In septic shock or in mixed forms of hypovolemia-associated shock, including bleeding, renal perfusion is limited by centralization to heart and central-nervous-system perfusion.

**Figure 1:**
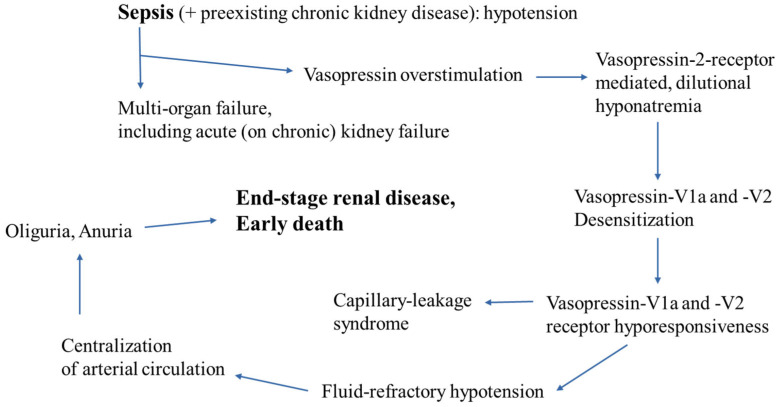
Role of acute kidney injury in vasopressin action during sepsis. Vasopressin, being released from the posterior lobe of the pituitary gland, stimulates renal vasopressin-2 receptor, which mediates water reabsorption via cAMP-activated aquaporin channels, and lowers the vascular–smooth muscle cell-mediated permeability of vessel walls. Hypothetically, both renal and vascular hyporesponsiveness to vasopressin contribute to sepsis-mediated hypovolemia in a vicious cycle [33].

A pre-existing renal insufficiency may limit the capacity of the body to counteract sepsis. In the early stages of sepsis, low blood pressure due to sepsis-associated arteriolar vasodilation is counteracted by tachycardia and increased vasopressin release due to baroreceptor activation, leading to increased renal water reabsorption. As for sepsis-induced hypoalbuminemia, the vascular underfilling aggravates the effect of arterial hypotension during sepsis. In these early stages of sepsis, fluid resuscitation may preserve renal function. In all forms of sepsis, the neurohormonal interplay between the heart and kidney plays an important role in maintaining organ function, adapting organ function to actual needs. Specifically, brain-type natriuretic peptide from the heart stimulates renal water and sodium excretion and serves as a laboratory surrogate value for acute or chronic heart failure. In addition, central-nervous-system and heart sympathoactivation, including catecholamine release from the adrenal glands, and the aforementioned vasopressin are activated during sepsis. As a consequence, dilutional hyponatremia occurs, thus rendering sepsis a common cause for hyponatremia in critically ill patients at hospital admission [31]. However, as shown in Figure 1, vasopressin responsiveness may be poor due to vasopressin receptor desensitization [2,32,33]

Thus, renal injury in sepsis may result from a combination of microvascular dysfunction, inflammatory processes, cytotoxic changes and impaired glomerular and tubular function [18,26,34,35]. Together, these pathophysiological mechanisms contribute to the development of AKI in sepsis.

## 4. Heart Failure and Sepsis

Intensive care medicine is faced with an increasing number of patients with more or less pronounced heart failure due to the age structure of today’s population and increasing life expectancy. These patients have limited ability to compensate for sepsis-related stress. They require more catecholamines to maintain stable hemodynamics and have a higher risk of mortality during their stay in the ICU. This observation has been confirmed recently by a prospective cohort study of 31,052 sepsis patients. In that study, a total of 974 patients with cardiac dysfunction, as determined by transthoracic echocardiography, were analyzed [36]. In the study, 136 patients (14.0%) had right ventricular dysfunction, 715 (73.4%) had left ventricular dysfunction, and 123 (12.6%) had biventricular dysfunction. More than 70% of patients with heart failure required intensive care, compared with 67.1% of patients without heart failure (*p* < 0.001). Among sepsis patients in the ICU, vasopressors were required in 33.1% to 51.2% of patients with cardiac dysfunction, compared to 23.0% of patients without cardiac dysfunction (*p* < 0.001). There were no significant differences in ICU or hospital length of stay. Mortality was highest in patients with RV dysfunction (27.2% vs. 17.7% without dysfunction).

However, sepsis itself can lead to reversible myocardial dysfunction (sepsis-induced cardiomyopathy, SCMP). This includes both systolic and diastolic dysfunction of the left and right ventricles [36].

**Figure 2:**
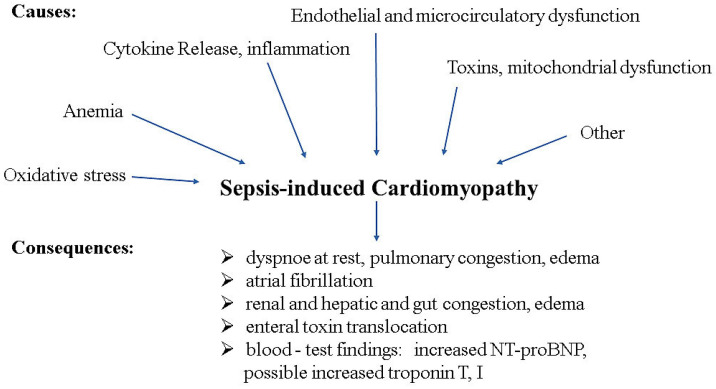
Main pathomechanisms contributing to and consequences of sepsis-induced cardiomyopathy.

The involvement of the heart in sepsis and its importance for the course and outcome are still often underestimated in everyday clinical practice, especially when systolic pump function is almost unimpaired and the focus of septic cardiomyopathy has shifted to diastolic dysfunction. The prevalence of septic cardiomyopathy varies widely, ranging from 10% to 70%, due to a lack of standardized definitions and numerous influencing factors such as gender, age, lactate levels and pre-existing cardiac dysfunction. However, in contrast to pre-existing heart failure, SCMP can be defined as acute cardiac dysfunction not associated with myocardial ischemia, comparable to dilated cardiomyopathy (DCM) and Takotsubo cardiomyopathy [37]. In particular, its reversible nature and pathophysiology (inflammatory or stress-related) are similar to Takotsubo cardiomyopathy, while its functional features (systolic dysfunction and ventricular dilation) are characteristic of DCM. Regarding the pathogenesis of SCMP, there is increasing evidence that an attenuated adrenergic response of cardiomyocyte filaments is the cause [38]. Supracellular mechanisms (a decrease in β1-adrenergic receptors and an increase in β3-adrenergic receptors) as well as intracellular mechanisms (a decrease in stimulatory G-proteins and an increase in inhibitory G-proteins) are likely to be of key importance [39,40]. These changes lead to reduced adenylate cyclase activity and reduced cAMP levels, which, in turn, limit inotropy. Figure 2 summarizes the pathomechanisms of SCMP.

The interaction of different pathomechanisms of SCMP and arrhythmias during sepsis is detailed below [41]:

1. Cytokine release and inflammation;

2. Metabolic changes in cardiomyocytes;

3. Endothelial and microcirculatory dysfunction;

4. Nitric oxide and oxidative stress;

5. Disturbance of calcium homeostasis;

6. Dysregulation of the autonomic nervous system.

### 4.1. Cytokine Release and Inflammation

Once again, over-activation of the innate immune system plays a key role and is the basis of the pathogenic processes in the development of septic cardiomyopathy mediated by DAMPs and PAMPs at the onset of sepsis, which triggers a cytokine storm (TNFα, IL-1, IL-6). The Toll-like receptors (TLR2 and TLR4) on the myocardial cell surface then recognize DAMPs (e.g., High-Mobility-Group-Protein B1) and PAMPs (e.g., bacterial LPS). Internal signaling pathways, e.g., the myeloid differentiation primary response 88 (MyD88) and the TIR-domain-containing adapter-inducing interferon-β (TRIF) pathway, are thereby activated and bind further enzymes, such as cJun N-terminal kinase (JNK), p38 MAPK and NF-κB, into the signaling pathway. The final result is the excessive release of so-called “immediate early genes” such as tissue factor, endothelin and proinflammatory cytokines, chemokines, adhesion molecules and enzymes, which drive a massive inflammatory reaction with tissue damage and destruction. A link to increased production of reactive oxygen species (ROS) and NO also exists, leading to oxidative stress and the disruption of mitochondrial function [41].

### 4.2. Metabolic Changes in Cardiomyocytes

In addition to the proinflammatory process described above, there are sepsis-related changes in the myocardium. The increased metabolic rate of the myocardium with a 30% increase in myocardial oxygen consumption also contributes to the development of septic cardiomyopathy. When septic myocardial dysfunction finally develops, oxygen consumption and the metabolic rate fall below the baseline values of healthy myocardium. In sepsis, fewer free fatty acids and ketones and less glucose are taken up by the cardiomyocytes. More damage to the myocardium is caused by altered activity of cardiac sympathetic nerve fibers and altered baroreflex responses. In addition, the process of phosphorylation and internalization has been demonstrated to result in the downregulation of β-adrenoceptors. This phenomenon leads to a reduction in the density of receptors present on the cell surface [42,43,44].

### 4.3. Endothelial and Microcirculatory Disorders and Myocardial Edema

Blood flow in the coronary arteries is increased during sepsis. Paradoxically, this increased coronary blood flow is associated with increased plasma troponin levels, which correlate with the severity of septic cardiomyopathy. Locally produced thromboxane A2 also reduces blood flow in the microcirculation. However, no myocardial necrosis was detected in patients who died with septic shock.

In contrast, experimental data indicate a clear septic change in the microcirculation in the myocardium, although this often varies regionally [45]. Regionally, the endothelium swells, fibrin deposits obstruct small vessels, and neutrophil granulocytes migrate into the interstitium of the myocardium and support the inflammatory reaction. Inflammatory mediators such as TNFα and IL-1β lead to activation and dysfunction of the endothelial cells. The endothelial barrier is weakened, causing fluid and plasma proteins to leak from the capillaries into the interstitium of the myocardium. This is enhanced by the adhesion molecules ICAM-1 and VCAM-1 and the binding of immune cells. Enlargement of the vascular leak causes myocardial edema. The increase in the microvascular fluid filtration rate and reduced removal of fluid via the lymphatic system lead to the accumulation of fluid in the interstitium of the heart muscle. This myocardial edema affects cardiac function by disrupting the balance of fluid flow and leading to deteriorated myocardial function and impaired myocardial compliance [45,46].

### 4.4. Nitric Oxide and Oxidative Stress

In cardiac muscle cells, three isoforms of nitric oxide synthase exist: eNOS, neuronal NOS (nNOS), and iNOS. While eNOS and nNOS constitutively produce small amounts of NO, iNOS is activated by inflammatory cytokines (such as IL-1β and TNFα) and produces large amounts of cytosolic NO. The activation of eNOS and nNOS modulates the responsiveness of cardiomyocytes to muscarinic cholinergic and beta-adrenergic receptors. Although the activation of iNOS is necessary to cause a significant decrease in myocyte contractile responsiveness to β-adrenergic agonists, it is not sufficient on its own. iNOS plays a key role in cardiac dysfunction through increased production of NO and reactive oxygen species (ROS) as well as decreased adrenaline sensitivity and calcium binding. This includes a reduced response of cardiac myofilaments to Ca^2+^ due to NO-induced overexpression of cyclic guanosine monophosphate (cGMP), as well as the involvement of NO in mitochondrial dysfunction and, ultimately, changes in pre- and afterload [47,48].

### 4.5. Impairment of Calcium Homeostasis

The alteration of calcium release in the context of septic cardiomyopathy is both complex and multifactorial. In sepsis, inflammatory mediators such as cytokines (TNFα, IL-1β) and NO lead to thr dysregulation of calcium release from the sarcoplasmic reticulum (SR). The function of the SERCA pump (sarcoplasmic/endoplasmic reticulum calcium ATPase) is responsible for the return of calcium to the sarcoplasmic reticulum and may be impaired. The contractile abilities of the heart muscle are restricted, resulting in reduced cardiac output. The impairment of cardiac output is exacerbated by increased activity of phospholamban, a protein that also regulates the SERCA pump [49].

Changes in the calcium sensitivity of actin and myosin are mediated by oxidative stress and proinflammatory mediators and lead to reduced cardiac output with perfectly normal calcium concentrations. Calcium itself is stored in the mitochondria and is then released from them as required. As part of their dysfunction, mitochondria lose the ability to buffer excess calcium, which, in turn, can lead to additional cell stress.

The increased production of NO activates guanylate cyclase. An increase in the cGMP level influences the calcium channels of the heart. Disturbed calcium homeostasis thus contributes significantly to the development and severity of SCMP and is an important target for potential therapeutic measures [50].

### 4.6. Dysregulation of the Autonomic Nervous System

In the context of sepsis, there is initially a disturbance in hemodynamics, as there is a disproportional increase in NO formation, primarily due to the increased induction of NO synthase (iNOS) via NFκB, but also due to cyclooxygenase-2 (COX-2) expression, leading to generalized vasoplegia, which is compensated for by increased activation of the sympathetic nervous system with the release of noradrenaline and adrenaline. The resulting increased inotropy, chronotropy and vasoconstriction can lead to sympathetic dysautonomia syndrome. In addition, patients receive significant amounts of externally administered catecholamines, particularly norepinephrine, especially in the initial period of sepsis. In the myocardium, such excessive stimulation of β-adrenergic receptors leads to inhibition of their expression by phosphorylation, and the number of receptors on the cell surface decreases. In addition, the transduction pathways are altered by reduced expression of Gs proteins and increased inhibitory activity of Gi proteins. This adrenergic storm itself can trigger cytokine production by cardiomyocytes, which, in turn, induces the release of downstream factors in the cascade of proinflammatory mediators (e.g., NO, oxygen radicals), leading to cardiomyocyte contractile limitation via mitochondrial respiratory chain dysfunction [51,52]. The catecholamine excess described above also leads to increased myocardial workload and oxygen consumption. While the septic heart is typically a net lactate extractor, the uptake of other substrates such as glucose, ketone bodies and free fatty acids is reduced, contributing to inefficient energy metabolism. During the course of septic shock, particularly in the setting of organ failure, both oxygen consumption and mitochondrial oxidative phosphorylation decrease, resulting in reduced ATP production. Since cardiac muscle has a much lower tolerance to hypoxia than skeletal muscle, these metabolic changes significantly impair cardiac function. Excess catecholamines therefore exacerbate this condition by increasing oxygen consumption and oxidative stress, which further promotes cardiac dysfunction. This pathogenesis and the positive results of β1-blockade in improving diastolic function and oxygen extraction through its negative chronotropic property are currently the basis for exploiting the beneficial effects of β1-blockade on myocardial function [39,40].

In summary, complex endothelial, metabolic, and immunologic abnormalities play a central role in septic cardiomyopathy, leading to impaired cardiac performance. In contrast to other forms of cardiomyopathy, myocardial ischemia is limited, although the impairment of myocardial blood flow does not play an insignificant role in the context of the inflammatory processes that otherwise occur.

## 5. Cross-Talk of Both Co-Morbidities

The presence of impaired cardiac function, regardless of its genesis, with its reduced myocardial contractility, leads to reduced blood flow to all organs, including the kidneys. In addition, cardiac dysfunction impedes venous flow, leading to the formation of edema, including intrarenal edema. Thus, reduced left ventricular ejection fraction, leading to inadequate renal perfusion, and renal congestion due to impaired venous return are the two main causes of AKI and the exacerbation of CKD in sepsis. The kidneys depend on adequate blood flow to maintain their filtration function. Inadequate blood flow due to centralization of the circulation in sepsis further impairs renal homeostasis and exacerbates renal failure. Conversely, AKI contributes to the deterioration of cardiovascular function. Toxic metabolites and proinflammatory mediators, especially cytokines, accumulate due to impaired renal function. These substances may enhance the direct toxic effect on the myocardium and thus contribute to SCMP.

Thus, the interaction between impaired cardiac function or SCMP and AKI is not an uncommon combination in septic patients and is based on a complex interaction characterized by systemic inflammatory processes, hemodynamic instability and microvascular dysregulation. The coexistence of SCMP and AKI is associated with significantly higher mortality and morbidity than the presence of either comorbidity alone [51].

Therefore, the early detection and treatment of SCMP and AKI are crucial to prevent a pathophysiological vicious cycle leading to increased mortality in sepsis patients.

## 6. Strategy for Diagnosis and Treatment

There are national and international guidelines for evidence-based diagnosis and therapy, which are updated on the basis of the latest literature [53,54]. However, patient-specific aspects and the interaction of existing organ functions are not adequately reflected in these guidelines. In addition, there is no evidence-based organ-specific treatment approach. Rather, treating individual organs needs to be considered in the overall management of sepsis. A patient-specific treatment approach therefore needs to be developed, as the treatment of sepsis in patients with existing congestive heart failure, cardiomyopathy and impaired renal function often has conflicting goals.

As supported by a high level of evidence, it is essential that the focus of infection be treated promptly and that appropriate antibiotics be administered as soon as possible after diagnosis. The 2016 Surviving Sepsis Campaign guidelines, updated in 2018, recommend lactate monitoring, blood cultures with calculated antibiotic administration, and the standard rapid administration of at least 30 mL/kg of intravenous crystalloid fluid in the 1-h initial resuscitation bundle to achieve a mean arterial blood pressure of 65 mmHg and serum lactate ≤2 mmol/L as a therapeutic goal [52,54]. The fluid management recommendation has an especially low level of evidence, but is currently used as part of the 1-h bundle as a quality criterion for assessing hospital-specific sepsis therapy [53]. In their retrospective analysis of 49,331 patients from 2014 to 2016, Seymour et al. were able to show that it is much more important to administer antibiotics within the 1-h bundle than to give the patient 30 mL/kg of IV crystalloid balanced fluid as quickly as possible [55]. Unless the mean blood pressure (MAP) has not increased to the target value of ≥65 mmHg after the initial fluid loading, vasopressors should be given within the first hour. This may lead to circulatory overload and worsen clinical status, especially in patients with impaired cardiac function due to reduced cardiac output.

A retrospective analysis of 598 septic patients, with a predominance of NYHA III patients (83.9%), rather supports the concept of individualized fluid administration and early use of catecholamines to avoid fluid overload. The analysis showed an optimal fluid requirement of 10–15 mL/kg patient weight during the first 3 h [56]. Prospective validation of these results in a clinical context is required. Clinical and technical options must therefore use fluid administration tailored to the needs of the individual patient. In general, it is important to prevent a further decline in SVR and cardiac contractility and to avoid fluid overload [57,58]. And this is precisely where the importance of personalized therapy tailored to the current cardiac performance lies. The underlying pathophysiological idea is the modified Frank—Starling mechanism, which is altered by a reduced cardiac output [59]. There is no evidence regarding the optimal mean blood pressure for maintaining adequate homeostasis. The sole evidence is that a MAP ≤65 mmHg in conjunction with a lactate ≥2 mmol/L should be avoided [1]. The optimal mean arterial blood pressure (MAP) is currently considered to be 75 mmHg ± 10 mmHg; higher values are associated with increased mortality or the occurrence of cardiac arrhythmias [57,58,60].

In the case of impaired cardiac function or when fluid requirements cannot be adequately met by infusions, the early use of vasopressor catecholamines with primarily vasoconstrictive α-adrenergic action, particularly noradrenaline (norepinephrine), is recommended. These substances are essential to maintain circulation and perfusion. In specific cases where additional positive inotropic support is required, such as pre-existing cardiomyopathy or sepsis-induced cardiomyopathy, the administration of β-adrenergic catecholamines, particularly dobutamine, could be important. Dobutamine may theoretically help stabilize the circulation by increasing myocardial contractility. A meta-analysis found that the use of dobutamine does not result in a significant survival benefit, even in patients with severe heart failure [61,62]. Dobutamine also causes vasodilation and decreases MAP, as well as increasing the risk of serious ventricular arrhythmias [63]. The inotropic effect may also be blunted in septic patients. If the chronotropic effect is maintained, the resulting tachycardia may cause a deterioration in oxygen balance without an increase in stroke volume (SV) [64]. Furthermore, the catecholamine epinephrine is associated with a higher incidence of refractory shock than norepinephrine, as demonstrated in randomized controlled trials [65].

However, if the shock is catecholamine-refractory, i.e., there is no adequate response to primary catecholamines and no additional positive inotropic support is required, the Sepsis Guideline recommends the use of vasopressin. The Surviving Sepsis Campaign (SSC) 2021 suggests the introduction of vasopressin when the norepinephrine dose is in the range of 0.25–0.5 µg/kg/min. Although the administration of vasopressin can significantly reduce the dose of norepinephrine, no direct survival benefit has been demonstrated in clinical trials to date [66]. However, a notable study by Gordon et al. showed that early use of vasopressin was associated with a reduced need for renal replacement therapy, suggesting a potential benefit in the management of sepsis-induced organ failure [67]. This finding needs to be validated in further prospective studies.

Recently, the vasoconstrictor peptide angiotensin II has been approved as a treatment option and investigated in a randomized controlled trial. In septic patients with a continuous norepinephrine dose of more than 0.2 µg/kg/min, angiotensin II was effective in raising mean arterial pressure (MAP) by more than 10 mmHg or to levels above 75 mmHg, but without a significant effect on the outcome. However, a further subgroup analysis showed an improvement in survival in patients who were on renal replacement therapy at the time of randomization.

Hypothetically, from a purely pharmacological perspective, patients taking an ACE inhibitor to self-medicate could be expected to benefit. However, to confirm the clinical relevance and long-term effect of angiotensin II in this patient population, this finding, which suggests a potential benefit in severe renal failure, needs to be further investigated in future prospective randomized trials as well [39,68,69,70].

Levosimendan and phosphodiesterase inhibitors have no scientific evidence regarding their use in the treatment of septic shock, with no current evidence of a beneficial effect on outcomes [71,72].

Finally, the use of mechanical heart support systems in the context of septic shock has not been adequately supported by the results of scientific studies. Due to the reversibility of septic cardiomyopathy, a mechanical support system can be used as a “bridge to recovery” on an individual basis [62,73].

Sepsis-related modulation of the beta-adrenergic system is a more recent concept. Short-acting, highly cardioselective β_1_-blockers such as esmolol or the even more potent landiolol have a limited effect on blood pressure. They have positive inotropic effects and stabilize heart function by controlling heart rate, and have been shown experimentally to have a positive impact on immunity, oxygen supply, metabolism and coagulation homeostasis [39,70].

The management of personalized fluid and vasopressor therapy to improve tissue oxygenation by increasing cardiac output requires the use of clinical and/or technical skills to assess the current clinical situation. The reduction in lactate levels, which may reflect improved tissue perfusion and is currently a recognized therapeutic goal, could be monitored to verify the efficacy of this therapy [74]. However, this parameter is not entirely uncriticized in terms of its clinical significance in septic patients [75]. Dynamic parameters such as pulse pressure variability, inferior vena cava compression and capillary refill time are currently preferred to static parameters such as central venous pressure, cardiac volume and pulmonary artery occlusion pressure for assessment in the context of cardiac function. Capillary refill time (CRT) has proven to be a very good and easy-to-perform test. In the Andromeda trial, it was found to be equivalent to lactate clearance with respect to 28-day mortality [76]. However, organ dysfunction based on the SOFA score at 72 h was significantly lower in the CRT optimization group (5.6 versus 6.6, *p* = 0.045). The other dynamic parameters, such as the passive leg raise test or stroke volume variability, were similarly positive when controlling for initial fluid therapy. In contrast, the sonographic measurement of inferior vena cava diameters during respiration was not shown to be reliable as compared to measurement of the extravascular lung water index through transpulmonary thermodilution [77]. However, this parameter is a relatively slow parameter as it increases only at later stages of sepsis, hinting at fluid overload [78]. Ultrasound is increasingly used in daily clinical practice. However, in the current context, it is only indicated for the assessment of cardiac function in patients with vasopressor-dependent septic shock. It helps to identify the shock type (left, right or biventricular) and differentiate other causes of cardiogenic shock. Parameters such as LVOT VTI (left ventricular outflow tract velocity–time integral), the E/e’ ratio (pulsed Doppler E wave ratio) and TAPSE (tricuspid annular plane systolic excursion) are also commonly used to objectify left and right ventricular function and identify high-risk patients. However, these require qualified staff, whereas the dynamic parameters already described, such as CRT, the passive leg-raising test or pulse pressure variation, can be performed and evaluated by less qualified personnel.

The monitoring of renal function is essential to assess renal–heart crosstalk. From a practical point of view, the assessment of urine output in relation to infusions and mean arterial pressure, as well as classic parameters such as serum creatinine, creatinine clearance and sodium, are needed to keep track of renal function during sepsis. As a novel parameter, vasopressin overstimulation can be tracked by serial measurements of copeptin, a stable surrogate of vasopressin [79]. Modern point-of-care parameters such as cystatin-C, proenkephalin A 119-159 (penKid) and TIMP2/IGFBP7 are becoming increasingly important to identify patients at high risk, particularly at an early stage. First studies show that Proenkephalin A 119-159 (penKid) is very promising as a sensitive marker for acute kidney injury (AKI) because it detects glomerular changes before serum creatinine and is not influenced by inflammatory mechanisms [80]. Presepsin, a biomarker released by microbially activated monocytes and macrophages, shows promising results as an indicator of sepsis and sepsis-associated AKI (SA-AKI). In addition, gene expression profiles, such as the recently identified AFM gene (afamin), as well as biomarkers from proteomics and metabolomics, provide new approaches for diagnosis and prognosis. TIMP2/IGFBP7 predicts the risk of severe AKI, whereas penKid is a functional marker of glomerular filtration, detecting early changes independent of tissue injury [81,82].

## 7. Looking Ahead to the Future

General therapy concepts based on current guidelines and the extensive standardization of initial therapy with the 1-h bundle or one or other supportive therapies (e.g., activated protein C) have not led to a real improvement in sepsis mortality [83]. Individualized concepts (precision medicine) to assess individual immune status are therefore increasingly favored and scientifically investigated [84,85]. Modern point-of-care diagnostic tools or data technologies (artificial intelligence, machine learning) could offer promising support for diagnostic and therapeutic decisions. In the future, this will enable better consideration of the various phases and individual manifestations of the dysregulated immune response to existing infection in the context of existing organ dysfunctions. However, until we have an effective tool for screening according to clinical criteria with a high level of specificity and sensitivity, it makes little sense to exclusively “reach for the stars” with the new technologies. If a serious infection is not adequately recognized, patients will continue to receive adequate diagnostics and initial therapy too late. Therefore, the need to search for an effective screening tool must not be underestimated in the scientific community. Decision support systems can then help determine the right therapy for each patient, tailored to their comorbidities and dysregulated immune response. Until clinically accepted digital decision support systems are available, however, the current concepts of initial therapy, which are only focused on intensive care symptom control (fluid management, calculated infection control), must be converted into precisely individualized intensive care medicine.

In this context, it seems equally important to ask whether mortality is still the correct primary endpoint of clinical trials when evaluating new methods and concepts. Some initial considerations have already been published as a basis for discussion. Diseases such as sepsis or severe traumatic brain injury are too complex and the patient population too heterogeneous to objectively determine the value of a single therapy.

## 8. Summary

Despite all the advances made in recent years, sepsis remains a challenge in intensive care medicine, notably because of the heterogeneity of the underlying a dysregulated immune response, but also because of the highly individualized patient population with many pre-existing organ dysfunctions. In particular, the kidneys and the heart may be the target organs of the dysregulated immune response in sepsis, and may thus be functio-nally compromised. Pre-existing organ dysfunctions may contribute significantly to the fatal outcome of sepsis. Both organ dysfunction and cardiorenal syndrome are interrelated and mutually reinforce each other in sepsis conditions, thus favoring multi-organ dysfunction and failure. On the other hand, organ-specific therapies for comorbid heart failure may be in conflict with general sepsis therapies, thus limiting the therapeutic options.

An early and differentiated therapy strategy based on an array of diagnostics, including cytokine levels and surrogate laboratory parameters of renal and cardiac dysfunction, hyponatremia as a surrogate for renal vasopressin action may help stabilize the circulatory situation and control the dysregulated individual inflammatory response. It is crucial to improve the prognosis of these patients, taking into account their pre-existing organ dysfunction. Modern data technology may yield further reliable patient data to monitor patients in real time to translate pathophysiology into modern, individualized precision medicine.
